# Functional Near-Infrared Spectroscopy Indicates That Asymmetric Right Hemispheric Activation in Mental Rotation of a Jigsaw Puzzle Decreases With Task Difficulty

**DOI:** 10.3389/fnhum.2020.00252

**Published:** 2020-06-30

**Authors:** Murat Can Mutlu, Sinem Burcu Erdoğan, Ozan Cem Öztürk, Reşit Canbeyli, Hale Saybaşιlι

**Affiliations:** ^1^Institute of Biomedical Engineering, Boğaziçi University, Istanbul, Turkey; ^2^Department of Medical Engineering, Acιbadem Mehmet Ali Aydιnlar University, Istanbul, Turkey; ^3^School of Sport Exercise and Health Sciences, Loughborough University, Leicestershire, United Kingdom; ^4^Department of Psychology, Boğaziçi University, Istanbul, Turkey

**Keywords:** fNIRS, lateralization, mental rotation, puzzle, DLPFC, prefrontal cortex

## Abstract

Mental rotation (MR) is a cognitive skill whose neural dynamics are still a matter of debate as previous neuroimaging studies have produced controversial results. In order to investigate the underlying neurophysiology of MR, hemodynamic responses from the prefrontal cortex of 14 healthy subjects were recorded with functional near-infrared spectroscopy (fNIRS) during a novel MR task that had three categorical difficulty levels. Hemodynamic activity strength (HAS) parameter, which reflects the ratio of brain activation during the task to the baseline activation level, was used to assess the prefrontal cortex activation localization and strength. Behavioral data indicated that the MR requiring conditions are more difficult than the condition that did not require MR. The right dorsolateral prefrontal cortex (DLPFC) was found to be active in all conditions and to be the dominant region in the easiest task while more complex tasks showed widespread bilateral prefrontal activation. A significant increase in left DLPFC activation was observed with increasing task difficulty. Significantly higher right DLPFC activation was observed when the incongruent trials were contrasted against the congruent trials, which implied the possibility of a robust error or conflict-monitoring process during the incongruent trials. Our results showed that the right DLPFC is a core region for the processing of MR tasks regardless of the task complexity and that the left DLPFC is involved to a greater extent with increasing task complexity, which is consistent with the previous neuroimaging literature.

## Introduction

In recent years, the number of studies conducted with functional near-infrared spectroscopy (fNIRS) has increased due to several advantages it has over other functional imaging techniques such as electroencephalography (EEG), functional magnetic resonance imaging (fMRI), and positron emission tomography (PET). fNIRS technology offers a more cost-effective and user-friendly alternative to fMRI and PET in measuring cerebral energy metabolism besides having the major advantage of being field-deployable. Furthermore, its portability allows recordings of the functional cerebral hemodynamics induced by cortical activation in daily life environments. Numerous studies have demonstrated the reliability of fNIRS in measuring cortical hemodynamic responses during various lateralized cognitive tasks involving language (Herff et al., 2013; Jasinska and Petitto, 2013; Sugiura et al., 2015; Fu et al., 2016), visual/visuospatial (Herff et al., 2013; Baker et al., 2018; Curtin et al., 2019) and mathematic processing (Herff et al., 2013; Artemenko et al., 2018). However, studies that have investigated visuospatial processing with fNIRS is limited in number, particularly for mental rotation (MR) tasks, the decoding of which might have crucial importance in the design of fNIRS based brain-computer interfaces.

Mental rotation is a spatial cognitive ability that involves mentally rotating two or three-dimensional objects. Since the development of the first mental rotation test by Shepard and Metzler (1971), mental rotation skill has been widely investigated by numerous research groups employing different behavioral and neuroimaging methods. While initial studies depended mostly on behavioral responses and usually measured reaction time as a metric of cognitive workload and difficulty (Shepard and Metzler, 1971; Cooper and Shepard, 1975), more recent studies have employed neuroimaging methods such as fMRI (Jordan et al., 2001; Halari et al., 2006; Seydell-Greenwald et al., 2017), fNIRS (Shimoda et al., 2008) and PET (Harris et al., 2000) to understand the neurophysiological processes underlying MR skill. In these studies, hemispheric dominance in mental rotation skill has been a major scope of the investigation, but the results still remain to be a matter of debate. While a majority of the studies claimed a right-hemispheric superiority in mental rotation with different imaging modalities (Ratcliff, 1979; Papanicolaou et al., 1987; Deutsch et al., 1988), others found a left-hemispheric dominance (Mehta and Newcombe, 1991; Alivisatos and Petrides, 1997) or a bilateral activation (Cohen et al., 1996; Vingerhoets et al., 2002; Hugdahl et al., 2006; Shimoda et al., 2008). While various factors may contribute to the discrepancy in these results, variations in task complexity and the resultant cognitive effort appear to be the first and foremost issue to be addressed. Mehta and Newcombe (1991) found that left hemispheric lesions affected the mental rotation of 3D-shapes, while Ratcliff (1979) and Ditunno and Mann (1990) found that right hemispheric lesions impaired the mental rotation of 2D-shapes. It is claimed that left-hemispheric activity increases with the complexity or difficulty of the task possibly because of two factors: applied rotation strategy (piecemeal vs. holistic) and involvement of other cognitive elements such as verbalization (Corballis, 1997). Corballis (1997) claimed that simpler shapes are being rotated in more holistic fashion, while more complex shapes are being rotated in more piecemeal fashion during which the subjects focus on certain features or attributes of the shapes. For the latter factor, increased task complexity requires various additional cognitive resources to be utilized during the task.

Since findings regarding the hemodynamic activation patterns observed during mental rotation tasks are not consistent in terms of lateralization, the present study aimed to assess whether MR elicits a lateralized neural response in the prefrontal cortex regions and, if so, where the hemodynamic activity is localized. Another aim was to investigate the effect of task difficulty on prefrontal cortex activation. Overall, the study was designed to show that fNIRS is reliably capable of capturing the cerebral hemodynamics during mental rotation tasks, and thus can be used in decoding of cognitive abilities. For these purposes, a novel mental rotation task based on jigsaw puzzle piece-template pairs with various categorical difficulty was designed, and fNIRS data were collected during execution of these tasks in a daily life environment rather than inside an MRI or PET apparatus. Several fNIRS studies have derived spatial and temporal features from hemodynamic responses during mental rotation tasks for fNIRS-Brain Computer Interface (BCI) applications (Herff et al., 2013; Banville et al., 2017; Qureshi et al., 2017). However, to the best of our knowledge, no fNIRS study has examined the spatial and temporal features of the cerebral hemodynamics of mental rotation tasks of varying difficulty at a group level in detail to reveal the underlying physiological mechanisms in the prefrontal cortex.

## Materials and Methods

### Participants

Fifteen healthy volunteer subjects, ages 21–35, participated in the study. One subject was excluded from analysis due to poor signal to noise ratio in fNIRS recordings. The remaining subjects were eight females and six males (11 right-handed and 3 left-handed). All subjects were informed before the experiment and gave written consent. The study was approved by the local ethical committee, Human Research Ethical Committee of Boğaziçi University.

### fNIRS Data Acquisition

**FIGURE 1:**
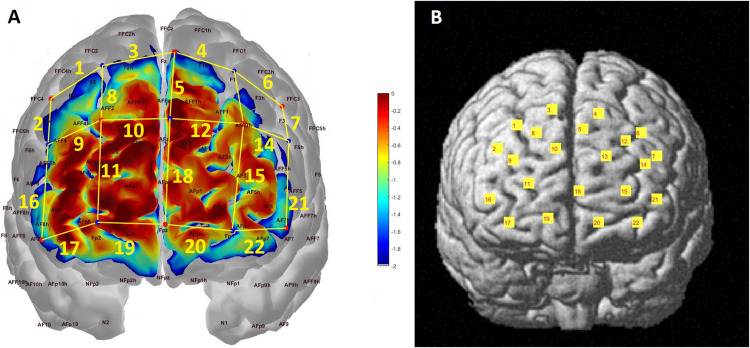
**(A)** Probe configuration and photon sensitivity profile. Each red dot corresponds to a light source while each blue dot represents a detector. Channels are formed between adjacent source-detector pairs and represented with yellow lines and labeled accordingly. The sensitivity of the probe to detecting brain hemodynamics is presented as a heat map in log10 units. **(B)** Channel locations projected onto a standard brain template in MNI space.

A NIRSport system (NIRx Medical Technologies, LLC, Berlin, Germany) with a set-up of 22 channels consisting of 8 light sources (emitting near-infrared light at 760 and 850 nm) and 7 detectors covering the frontal cortex was used to collect hemodynamic data (Figure 1A). The sampling rate of the signal was 7.8125 Hz and the source-detector distance was 3 cm. Probe sensitivity profile was computed with the Atlas Viewer toolbox (Aasted et al., 2015) of Homer2 Software (Huppert et al., 2009). AtlasViewer provides wavelength-specific sensitivity maps of photon propagation for each. In Figure 1A, the optical sensitivity profile of the probe is mapped on a standard brain template with a 10/5 global EEG electrode system. The sensitivity of the probe for detecting brain hemodynamics was calculated with the method explained in Aasted et al. (2015) and presented as a temperature plot ranging from 0 (red) to -2 (blue) in log10 units. The channel locations are coregistered onto a standard brain in MNI space using the NIRS_SPM toolbox in Figure 1B (Singh et al., 2005; Jang et al., 2009; Ye et al., 2009). The percentage of Brodmann areas covered by each channel was computed with the spatial registration toolbox of NIRS_SPM according to the Rorden’s brain atlas (Rorden and Brett, 2001) and are presented in Table 1.

**TABLE 1:** The MNI coordinates and the percentage of Brodmann areas covered by each projected channel.

Channels	MNI coordinates	Brodmann’s areas	Percentage of overlap
	
	x y z		
CH1	29 46 45	BA9	0.99
		BA46	0.01
CH2	41 49 32	BA9	0.08
		BA45	0.2
		BA46	0.72
CH3	11 48 52	BA8	0.16
		BA9	0.84
CH4	−12 48 50	BA8	0.05
		BA9	0.95
CH5	−4 57 41	BA9	0.89
		BA10	0.11
CH6	−33 44 40	BA9	0.64
		BA46	0.36
CH7	−42 48 28	BA45	0.42
		BA46	0.58
CH8	20 56 41	BA9	0.96
		BA46	0.04
CH9	31 60 27	BA9	0.01
		BA10	0.28
		BA46	0.71
CH10	11 66 31	BA9	0.21
		BA10	0.79
CH11	23 71 14	BA10	1
CH12	−27 53 37	BA9	0.50
		BA46	0.50
CH13	−16 65 28	BA9	0.16
		BA10	0.79
		BA46	0.05
CH14	−36 56 25	BA10	0.01
		BA46	0.99
CH15	−27 68 10	BA10	0.89
		BA11	0.11
CH16	44 60 6	BA10	0.54
		BA46	0.46
CH17	34 67 −6	BA10	0.26
		BA11	0.61
		BA46	0.03
		BA47	0.10
CH18	−2 69 11	BA10	1
CH19	13 73 −4	BA10	0.50
		BA11	0.50
CH20	−13 73 −5	BA10	0.37
		BA11	0.63
CH21	−43 57 5	BA10	0.37
		BA46	0.63
CH22	−32 66 −6	BA10	0.32
		BA11	0.54
		BA46	0.04
		BA47	0.10

### Experimental Design

**FIGURE 2:**
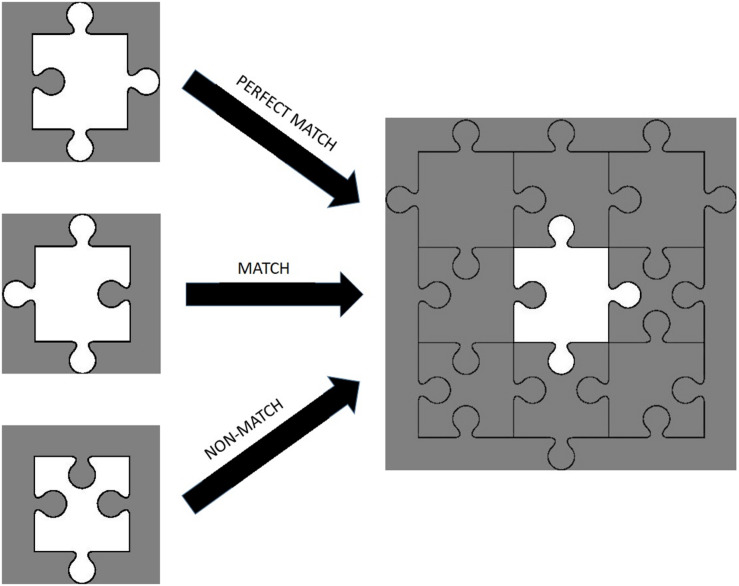
Examples of perfect match, match, and non-match trials.

**FIGURE 3:**
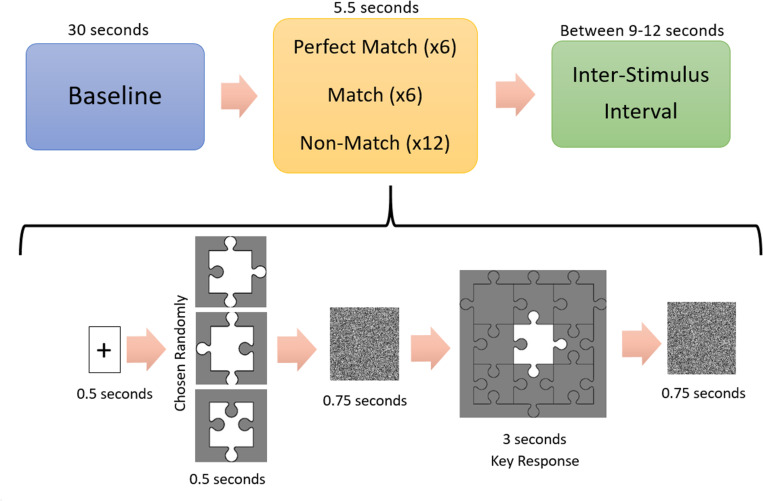
Experimental procedure. A 30 s long-baseline recording was followed by a 0.5 s long fixation point shown in the center of the computer screen. After turning off the fixation point, subjects saw a randomly chosen jigsaw puzzle piece, followed by 0.75 s long white noise masking image. Subsequently, the puzzle template was shown on the screen for 3 s during which the subjects had to give a “Yes” (“Matched”) or “No” (“Unmatched”) answer. Another 0.75 s long white noise masking image was followed by an inter-stimulus interval randomly chosen from the range of 9–12 s.

The study was carried out in a silent and dimly lit room. The temperature of the room was controlled with an air conditioning system and set approximately at 23°C. Subjects sat in front of a computer screen with a 50 cm eye-to-screen distance and all experimental cues were presented on a 24-inch computer monitor. After the acquisition of demographic data, the experimental protocol was explained in detail to each subject. During the experiment, subjects had to decide whether a puzzle piece shown on the screen could fit the gap in the middle of a puzzle template, shown after the puzzle piece, by pressing either the “S” or “K” key on the keyboard, indicating a “Yes” (“Congruent”) or “No” (“Incongruent”) answer respectively. There were three conditions: (1) Perfect Match (PM) condition in which the piece can fit into the template without requiring a mental rotation, (2) Match (M) condition in which the piece would fit into the template only if the subject mentally rotated the piece, and (3) a Non-Match (NM) condition in which the piece could not fit into the template (Figure 2). Each subject made decisions for 24 trials consisting of 6 PM, 6 M, and 12 NM conditions presented in random order. All of the Perfect Match and Match trials formed a “congruent” condition and required a “Yes” answer, while Non-Match trials formed an “incongruent” condition requiring a “No” answer. Each experiment began with a baseline fNIRS recording of 30 s, after which the subject pressed the “Space” key on the keyboard to start the experiment. At the beginning of each trial, a plus sign (“+”) was presented for 500 ms to briefly alert the subject to the onset of a new trial. Then, a puzzle piece was presented for 500 ms, followed by a white noise masking image presentation for 750 ms to eliminate the after-image effect of the puzzle piece. Subsequently, the template was shown for 3 s during which the subject had to give a “Yes” or “No” answer by pressing “S” or “K” on the keyboard, respectively. Afterward, a white noise masking was shown for another 750 ms. All of the visual cues and images were rendered at the center of the screen in order to eliminate a possible visual field effect. The total duration of a trial was 5.5 s and the inter-trial interval was set randomly for each trial, ranging from 9 to 12 s, in order to prevent subjects’ anticipatory hemodynamic responses (Figure 3). The experiment was designed with PsychoPy 3.0.6 (Peirce et al., 2019). Reaction times and accuracy rates for each trial were collected as behavioral data with PsychoPy software. fNIRS data were collected continuously during the experiment.

### Data Preprocessing and Feature Selection

Data preprocessing was performed with a combination of nirsLAB (a free analysis software that comes with the NIRSport system), Homer2 and custom-made scripts written in MATLAB R2017B. Raw intensity data were first visually examined in nirsLAB. Then, the coefficients of variation (CV) method (Hocke et al., 2018; Pfeifer et al., 2018) was employed to eliminate the channels with poor signal quality. The threshold for CV was set to 7.5% as used by Hocke et al. (2018). A preprocessing pipeline based on HOMER2 functions was constructed. The raw intensity data were converted into optical density (OD) data with hmrIntensity2OD function. Data segments that included motion artifacts were identified by using hmrMotionArtifact.m function with the following parameters: tMotion = 0.5, tMask = 1, STDEVthresh = 10, and AMPthresh = 1. PCA filtering was applied for motion artifact removal by using hmrMotionCorrectPCA.m with the parameter nSV set to 0.8. Motion corrected OD data were filtered with a third-order Butterworth bandpass filter with high and low cut-off frequencies of 0.01 and 0.2 Hz, respectively. Lastly, oxygenated hemoglobin (HBO) and deoxygenated hemoglobin (HBR) concentrations were calculated from filtered optical density data by using hmrOD2Conc.m function. Only HBO data were used in the analysis as it was shown that HBO is a more reliable indicator of cortical activation (Watanabe et al., 2002; Dravida et al., 2017).

### Data Analysis

#### Behavioral Data Analysis

Accuracy was calculated by dividing the number of successfully answered trials by the total number of trials for each subject. In the reaction time dataset, for the trials during which the subject could not give a valid answer within the 3 s response period, reaction time was set at 3 s, the maximum. Due to the unbalanced number of trials among conditions, we calculated the average reaction time and accuracy per condition for each subject. Therefore 14 × 3 matrices (number of subjects × conditions) were created for reaction time and accuracy separately and were subjected to one-way repeated measures ANOVA.

#### fNIRS Data Analysis

For each trial, 14 s long block segments were created which spanned a 2 s pre-stimulus baseline interval and a 12 s long duration after the onset of stimulus presentation. Each segment was detrended and classified as a Perfect Match, Match or Non-Match condition trial. We defined a new parameter, called Hemodynamic Activity Strength (HAS), for hemodynamic time series as in the following: First, the time point corresponding to the maximum concentration value between 4.5 and 12 s period after the onset of a trial is determined. Then, a mean peak value (MPV) is calculated within a window, which spans 1.5 s before and after that peak. The mean value of the 2 s pre-stimulus baseline window is calculated (MB) and subtracted from the MPV. Lastly, this value is divided by the standard deviation of the pre-stimulus baseline window.

##### Hemispheric lateralization analysis

At the single subject level, whether a channel was deemed significant was tested for each condition as follows: (1) Single-trial HAS parameter of each trial (n = 6 for PM and M conditions; n = 12 for NM condition) was computed separately for each condition and channel with the abovementioned method. (2) One sample student t-tests were performed on the vector of HAS parameters belonging to each condition and channel separately. (3) Channels having statistical significance (p < 0.05) were considered as active channels.

Hemispheric Lateralization (HL) parameter was calculated for each subject and condition with the following formula:

$${\text{HL}}_{s\,u\,b,c\,o\,n}=\frac{N_{R\,C}-N_{L\,C}}{N_{C}}$$

where *N*_*RC*_ denotes the number of active channels in the right hemisphere; *N*_*LC*_ denotes the number of active channels in the left hemisphere; *N*_*C*_ is the total number of channels in a hemisphere (i.e., *N*_*C*_ equals to 10); and HL_*sub*,*con*_ denotes the hemispheric difference parameter for a subject and condition. With this formula, the hemispheric lateralization parameter is normalized between -1 and 1, where positive values denote right hemispheric lateralization and the negative values denote left hemispheric lateralization.

To assess whether there is a statistically significant hemispheric lateralization in a condition, 14 × 1 vectors of hemispheric lateralization parameters belonging to each condition were subjected to a one-sample student *t*-test. Furthermore, to assess whether there is a difference between conditions in terms of lateralization, hemispheric lateralization vectors were subjected to one-way ANOVA with the condition as the main effect.

##### Hemodynamic activity analysis

To localize the hemodynamic activity, group level HAS parameter was computed as follows: (1) For each channel and subject, the trial time series of each condition were block averaged, resulting in a single time-series data for each condition and channel in a subject (see section fNIRS Data Analysis). (2) A HAS parameter was calculated on this block-averaged time series with the abovementioned method. (3) Lastly, 14 × 3 matrices (subject × condition) were generated with group level HAS parameters for each channel.

To localize the statistically significant hemodynamic activity at the group level, one sample student *t*-test was performed on group level HAS parameter separately for each channel and condition. To assess contrasts among different conditions, a channel based one-way repeated measure ANOVA was performed with the condition as the main effect. *Post hoc t*-tests with Bonferroni correction were performed for pairwise comparisons for the following contrasts: Match > Perfect Match, Non-Match > Perfect Match and Non-Match > Match. Thresholded t-statistics depicting significant activation were mapped onto a standard head template for each condition and contrast. Including channels as a main factor in ANOVA analyses should be avoided in fNIRS studies because of the systematic bias caused by variations in optical properties of different brain regions, as suggested in the works of Yanagisawa et al. (2010) and Kujach et al. (2018). Thus channel was not included as a main factor in ANOVA. All statistical analyses were conducted with IBM SPSS Statistics 2015 and MATLAB R2017B.

## Results

### Behavioral Data

Reaction time and accuracy data are summarized in Table 2.

**TABLE 2:** Summary of the behavioral data in mean ± standard error.

	Perfect match	Match	Non-match	Overall
Accuracy	0.952 ± 0.102	0.941 ± 0.106	0.964 ± 0.063	0.952 ± 0.09
Reaction time (s)	1.146 ± 0.327	1.394 ± 0.486	1.21 ± 0.287	1.25 ± 0.382

#### Reaction Time

We found a significant difference among conditions in terms of reaction time [*F*(2, 39) = 4.931, *p* = 0.015]. Bonferroni corrected *post hoc* analysis of paired *t*-test results showed that reaction times for the Match trials were significantly longer than those for the Perfect Match trials (*p* = 0.02), while no significant difference was found between the Non-Match and the Match (*p* = 0.272) or between the Non-Match and the Perfect Match (*p* = 0.99) conditions, even though Non-Match required slightly longer reaction times than Perfect Match condition.

#### Accuracy

One way repeated measure ANOVA results did not show any significant difference among conditions for accuracy data [*F*(2, 39) = 0.236, *p* = 0.791].

#### Reaction Time vs. Accuracy

We calculated Pearson’s correlation between accuracy and reaction time for each condition. A negative correlation between the two behavioral metrics was found for each condition but only Match (*p* = 0.012, *R* = −0.648) and Non-Match (*p* = 0.026, *R* = −0.591) conditions surpassed a statistically significant threshold while Perfect Match condition did not (*p* = 0.165, *R* = −0.393).

### fNIRS Data

#### Hemispheric Lateralization Analysis

Hemispheric lateralization data are listed in Table 3. One sample student *t*-test results showed that Perfect Match condition had a statistically significant right hemispheric lateralization (HL = 0.071 ± 0.029, *p* = 0.027), while Match (HL = 0.029 ± 0.049, *p* = 0.566) and Non-Match (HL = 0.036 ± 0.064, *p* = 0.588) conditions did not present statistical significance even though they show a right hemispheric lateralization. Besides, 57% of the total number of active channels from all subjects were located on the right hemisphere in Perfect Match condition, while only 39% of active channels were located on the left hemisphere. One way repeated measure ANOVA revealed that there was no significant difference among conditions in terms of hemispheric lateralization [*F*(2, 39) = 0.22, *p* = 0.806].

**TABLE 3:** Hemispheric Lateralization (HL) parameter of each subject.

	S1	S2	S3	S4	S5	S6	S7	S8	S9	S10	S11	S12	S13	S14	Mean ± Std
*PM*	0.2	-0.1	0	0	0	0	0	0.1	0	0.1	0.1	0.3	0.1	0.2	0.071 ± 0.029
*M*	0	0	0	0.3	0	0.3	-0.2	0	0	0	0.4	-0.1	-0.2	-0.1	0.029 ± 0.049
*NM*	-0.1	-0.2	0.4	-0.3	0.3	0.1	0.1	0.2	0.3	-0.1	-0.1	0.2	0.1	-0.4	0.036 ± 0.064

*Positive values indicate right lateralization and negative values indicate left lateralization.*

#### Hemodynamic Activity Analysis

**FIGURE 4:**
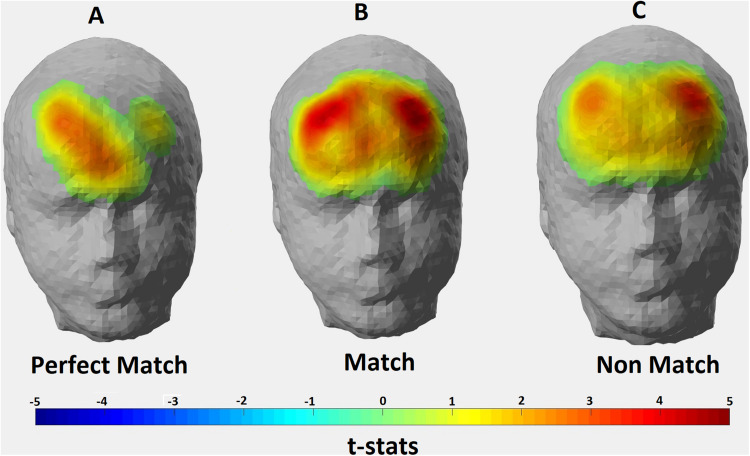
Hemodynamic activity strength parameter map for **(A)** Perfect Match; **(B)** Match; and **(C)** Non Match conditions. Channels depicting statistically significant activation (*p* < 0.05) were mapped onto a standard head model with their thresholded t-statistics score for each condition.

The *p*-values obtained from one-sample *t*-tests for each channel showed that significant hemodynamic changes occurred in 8 out of 22 channels for Perfect Match condition; 18 channels for Match condition; and 19 channels for Non-Match condition (*p* < 0.05). Figure 4 depicts the thresholded t-statistics parameter for each condition on a head model. In Perfect Match condition, neural activity was localized mainly in the right hemisphere (2 left, 5 right, 1 medial) while a bilateral neural activity was observed during Match (9 left, 8 right and 1 medial) and Non-Match (9 left, 8 right and 2 medial) conditions (Table 4).

**TABLE 4:** Significantly active channels and corresponding brain regions for each condition at the group level.

		PM	M	NM
Left	# of active channels	2	9	9
	Channel #	14, 20	4, 6, 7, 12, 13, 14, 15, 21, 22	6, 7, 12, 13, 14, 15, 20, 21, 22
Medial	# of active channels	1	1	2
	Channel #	18	18	5, 18
Right	# of active channels	5	8	8
	Channel #	1, 9, 10, 11, 19	2, 3, 8, 9, 10, 16, 17, 19	1, 2, 9, 10, 11, 16, 17, 19

**FIGURE 5:**
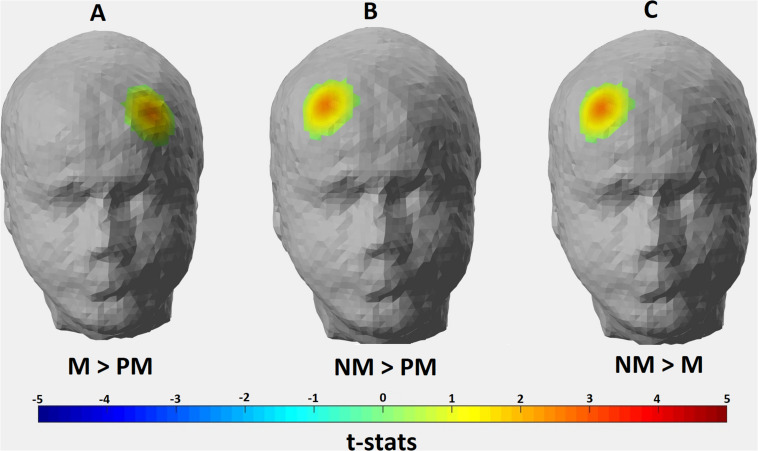
Hemodynamic activity strength parameter map depicting the contrasts for **(A)** Match > Perfect Match; **(B)** Non Match > Perfect Match; and **(C)** Non Match > Match conditions. Channels depicting statistically significant contrast (*p* > 0.05) were mapped onto a standard head model with their thresholded t-statistics score for each condition pair.

One-way repeated measure ANOVA revealed significant hemodynamic activity differences among conditions in two channels: Channel 9 [*F*(2, 26) = 6.500, *p* = 0.024] and Channel 14 [*F*(2, 24) = 4.059, *p* = 0.039]. These channels correspond to the right Brodmann Area (BA) 9, 10, 46, and left BA 10, 46, respectively. However, the majority of the signal collected by these channels come from right and left DLPFC, respectively (Table 1). *Post hoc t*-tests with Bonferroni correction showed that HAS of Match condition was stronger than Perfect Match condition only in the left DLPFC (*p* = 0.014); HAS of Non-Match condition was stronger than Match condition only in the right DLPFC (*p* = 0.005); and HAS of Non-Match condition was stronger than Perfect Match condition in only right DLPFC (*p* = 0.004) (Figure 5).

## Discussion

The aim of this study was to investigate the neural activity lateralization in the prefrontal cortex in response to mental rotation tasks with varying complexity and to demonstrate the reliability of fNIRS for capturing the neurophysiological mechanisms underlying mental rotation. For this purpose, a novel mental rotation task based on mentally fitting a jigsaw puzzle piece into a template was designed. Our results demonstrated a right hemispheric activation in all mental rotation tasks while recruitment of the left hemisphere with increasing task complexity.

### Behavioral Data

Assuming task difficulty is accompanied by longer reaction times as demonstrated by Shepard and Metzler (1971), the reaction time data revealed task difficulty as Match > Non-Match > Perfect Match. Perfect Match condition requires no mental rotation of the puzzle piece, thus it shows similarity to simple delayed match-to-sample task (DMTS). However, for the Match and Non-Match conditions, subjects needed to recruit further cognitive processes such as mental rotation, inner speech, and verbalization to make a judgment. Therefore, it can be assumed that in perfect match trials subjects only maintain the relevant information, but in the other two conditions, they had to manipulate it as well. The maintaining and manipulating processes will be discussed later in this report.

High accuracy rates (>%94 ± 1.5) observed in all three conditions indicated that all subjects actively participated in the tasks (Table 2). Even though no statistically significant difference was found in terms of the accuracy values among the three conditions, the Match condition had the lowest accuracy rate (%94.1 ± 2.8) suggesting that it was the most cognitively demanding task. On the other hand, negative correlations between reaction time and accuracy for all three conditions indicated that increased reaction time was associated with increased task difficulty (Shepard and Metzler, 1971; Cooper and Shepard, 1975).

### fNIRS Data

When the hemodynamic activity localization was analyzed at the group level for three conditions in terms of hemispheric lateralization parameter, a right hemisphere dominant activation was found for the Perfect Match condition whereas bilateral activations were found for Non-Match and Match conditions (Figure 4). Right DLPFC was found to be activated in all three conditions which was in accordance with many studies reporting right hemispheric activity during mental rotation tasks (Ditunno and Mann, 1990; Corballis, 1997; Harris et al., 2000; Hugdahl et al., 2006). Subjects had to keep the shape of the puzzle piece in their mind until the template is shown and manipulate it where necessary in order to arrive at a judgment. Therefore, visual working memory is essential for all three conditions. It is shown that right DLPFC is involved in visual working memory processes (Leung et al., 2002; Slotnick and Moo, 2006; Daniel et al., 2016) which was supported by our findings. As mentioned above, perfect match trials have a similarity with the simplest form of DMTS tasks. Daniel et al. (2016) described this type of DMTSs as having three phases: cue, delay, and response. In this type of DMTSs, subjects need only to maintain the relevant information in their visual working memory without further manipulation. Thus, activating only the right DLPFC might provide adequate neural sources for solving the perfect match trials. On the other hand, in match and non-match conditions, subjects had to manipulate the information stored in visual working memory in order to arrive at the correct answer. D’Esposito et al. (1999) demonstrated that mental tasks requiring the manipulation of the information stored in working memory are more difficult than tasks requiring only maintaining the relevant information. It is likely that the recruitment of left-hemispheric resources during Match and Non-Match conditions was because of the additional mental strategies (manipulations) employed as the task becomes more difficult. Feedback from subjects revealed that they tended to employ complex strategies during Match and Non-Match conditions such as focusing on local attributes (i.e., number and position of the protrusions) of the shapes rather than global attributes, and/or verbalization and inner speech. Multiple studies have also shown left hemispheric dominance in the processing of local features of complex shapes while right hemispheric dominance in processing global features regardless of the stimuli type (Christie et al., 2012; Brederoo et al., 2017). Language related brain activities are mostly localized in the left hemisphere (Frost et al., 1999) and inner speech is known to be regulated by the left hemisphere (Scholkmann et al., 2013). Another study by Hugdahl et al. (2006) suggested that verbalization might be one of the strategies used by subjects during mental rotation tasks and may explain the left-hemispheric activity. Significantly active channels found in the left hemisphere for Match and Non-Match conditions cover the BA 8, 9, 45, and 46 which are known to be involved in language processing. Hence, mental strategies deployed by subjects during Match and Non-Match tasks might explain the bilateral activation found in the prefrontal regions (Figure 4).

The location of the significant activation found for Match > Perfect Match contrast corresponds to the left DLPFC (Figure 5). As mentioned above, subjects used additional mental strategies (i.e., verbalization/inner speech) in Match condition compared to the Perfect Match condition due to the increased complexity of the task. It was shown that left DLPFC is involved in the processing of various linguistic tasks (Hugdahl et al., 1999; Abrahams et al., 2003). Hugdahl et al. (2006) also claimed that verbalization strategy might be the reason for left-hemispheric activity during the processing of mental rotation tasks. Therefore, we assume that stronger left DLPFC activity in Match condition compared to Perfect Match condition in our experimental design might be due to the use of inner speech and verbalization of local attributes of the stimuli in order to complete the Match trials. For the contrasts depicting Non-Match > Match and Non-Match > Perfect Match, the location of significant activation covered DLPFC of the right hemisphere (Figure 5). A stronger activation localized in the left hemisphere would be expected for Non-Match > Perfect Match and Match > Non-Match contrasts due to the increasing task complexity as revealed by behavioral metrics. However, a dominant right hemispheric activity observed in Non-Match compared to the other two conditions leads us to interpret the results from another perspective. In the Non-Match condition, subjects were exposed to incongruent stimuli and they were expected to correctly reject the unmatching piece-template pair. It is possible that this correct rejection mechanism may require strong conflict monitoring. Chevrier et al. (2007) used event-related fMRI to investigate the neural response for successful and unsuccessful inhibition of incongruent trials during the Stroop test and found that the right DLPFC is one of the active regions. A meta-analysis of Go/No-Go tasks carried out by Simmonds et al. (2008) also demonstrated that the right DLPFC is one of the brain regions found to be in association with successful inhibitions of No-Go trials. In our task, subjects had to recognize that the piece could not fit into the template in the Non-Match condition in contrast to the Perfect Match and Match conditions, thus replying with a “No” response in order to successfully reject the trial.

### Top-Down vs. Bottom-Up Modulation

In a meta-analysis review, Tomasino and Gremese (2016) explored the impact of the employed strategy (i.e., top-down modulation) and stimulus type (i.e., bottom-up modulation) on brain activation during mental rotation tasks. They included studies where the specific motor or visual imagery strategy instructions were given at the onset of the experiment. Their findings indicated that the mental rotation network included activations in the inferior frontal and middle frontal gyrus bilaterally, regardless of the stimulus type and type of strategy followed.

Our results indicated that the right DLPFC activation was common for all trial types. Right DLPFC activation is associated with visual working memory processes and may not necessarily be influenced by the type of strategy. As previous literature suggests (Corballis, 1997; Hugdahl et al., 2006), subjects may also employ secondary cognitive processes during mental rotation tasks such as local feature processing and inner speech, which are known to be regulated by left DLPFC resources. We propose that the bilateral activations observed during Match and Non-Match conditions in our experimental design are likely to be related to the use of secondary cognitive processes. It should be noted that Tomasino and Gremese (2016) excluded the studies in which the subjects were not instructed with a specific mental rotation strategy. In our study, we did not give any specific instructions to our subjects on whether to follow motor or visual imagery strategies at the onset of the experiment. Nonetheless, regarding the top-down regulation consensus reported in Tomasino and Gremese (2016), we may speculate that the bilateral DLPFC activation observed during the match and non-match conditions might be due to the usage of motor imagery-based strategy in addition to the abovementioned secondary cognitive processes.

Regarding the frontal cortex regions for mental rotation of non-bodily objects, Tomasino and Gremese (2016) reported activation in inferior frontal gyrus bilaterally and middle frontal gyrus. Our design included only non-bodily stimuli. Bilaterally activated frontal regions observed during Match and Non-Match conditions are in accordance with the findings of Tomassino et al. (2015). Our findings indicate that the left hemispheric activation increases with task difficulty for non-bodily stimuli.

### Recommendation for Future Work

In our protocol, the Match condition was the most cognitively demanding condition as supported by both behavioral and hemodynamic metrics (Table 2 and Figure 4). Post-experimental feedback from subjects suggested that their decision-making strategy involved counting the number of protrusions and recesses and/or a mental rotation strategy involving the dynamic spatial transformation of the puzzle piece. Seepanomwan et al. (2015) stated that the task difficulty increases with the number of distinctive features of stimuli. There are two distinctive features in our stimulus design: (i) the number of local features (i.e., protrusions and recessions), and (ii) their orientation. During the non-match conditions, checking either of the distinctive features would be sufficient to arrive at a decision. Subjects may prefer either counting the local features or rotating the puzzle piece. The Match conditions were more challenging than the Non-Match conditions because the subjects had to check both distinctive features, as checking only the number of local features would not be sufficient to make a correct decision (e.g., both the piece and the template may have two protrusions and recesses, yet the protrusions and recesses might be on the consecutive edges for the piece, while they might be located on the opposite edges for the template. Therefore, they had to check the second distinctive feature as well.). However, we should note that more challenging protocols involving match and non-match conditions can also be designed where it will be necessary to consider a mental rotation in all non-match trials (Seepanomwan et al., 2015). In future designs, different strategies might be employed to make the non-match condition more cognitively demanding, such as using unusual rotation angles (e.g., 15, 145, 20, 220 degrees) instead of multiples of 90 degrees, designing the puzzle piece and template with asymmetrical distinctive features.

Removal of instrumental, systemic, and motion artifacts in fNIRS signals generally consist of a combination of three steps: (1) motion correction; (2) bandpass filtering; and (3) low-frequency systemic noise removal. While numerous detailed signal processing methods have been developed for each step, there is still no consensus in literature for a standardized preprocessing pipeline (Pinti et al., 2019). Several methods have been proposed in the literature to eliminate the common systemic noise in fNIRS signals. Regarding data-driven approaches, global signal regression (Carbonell et al., 2011; Hocke et al., 2018), time-delayed global signal subtraction (Erdogan et al., 2016), ICA (Kohno et al., 2007; Santosa et al., 2013), and PCA based methods (Zhang et al., 2005) have been the most widely used methods. However, modeling systemic effects with regressors derived from short separation channel measurements that monitor only scalp hemodynamics are considered as the gold standard for global noise removal (Saager et al., 2011). Data-driven methods have limitations and the potential to distort the signals of interest which are extensively reviewed in Hocke et al. (2018) and Pinti et al. (2019). Our system did not have short separation channels for monitoring scalp hemodynamics. Hence; we decided to use minimal preprocessing practices to avoid possible signal distortion. For future work, we plan to compare the impact of the data-driven noise removal strategies with short channel removal methods for a better understanding of the noise characteristics of the signals collected from the prefrontal cortex region during cognitive tasks. We also plan to monitor frontal and parietal areas with a system equipped with a higher number of channels to investigate functional connectivity measures among different brain regions.

## Conclusion

Our findings indicate that the right DLPFC is one of the core brain regions activated during mental rotation regardless of the task difficulty. Our results also showed that left hemisphere activity becomes profound with increased task difficulty and the fact that the left hemispheric involvement does not depend on the dimension (2D or 3D) of the stimuli but on task difficulty. The present study showed that fNIRS is a reliable neuroimaging method to be utilized in mental rotation studies, or in general lateralization studies. It is of vital importance to show that fNIRS technology can be used in assessing various cognitive skills for two reasons. First, it allows for investigating the brain dynamics during cognitive tests in daily, natural environments rather than the noisy, stress-inducing artificial MR or PET settings. Secondly, due to its great ease of use when compared with EEG, fNIRS is a good candidate for new generation BCI systems.

## Data Availability Statement

The datasets generated for this study are available on request to the corresponding author.

## Ethics Statement

The studies involving human participants were reviewed and approved by the Human Research Ethical Committee of Boğaziçi University. The patients/participants provided their written informed consent to participate in this study.

## Author Contributions

MCM, SE, RC, and HS designed the experiments. MCM and OÖ carried out the experiments. MCM and SE analyzed the data, wrote the manuscript, and prepared the figures. All authors reviewed the manuscript.

## Conflict of Interest

The authors declare that the research was conducted in the absence of any commercial or financial relationships that could be construed as a potential conflict of interest.
